# Exploring the impacts of high-speed rail on technology-intensive manufacturing: the case of the Yangtze River Delta region, 2007–2016

**DOI:** 10.1038/s41598-024-72611-9

**Published:** 2024-09-19

**Authors:** Xiuxin Ma, Anton Van Rompaey, Will W. Qiang, Ben Derudder

**Affiliations:** 1 Public Governance Institute, KU Leuven, Leuven, Belgium; 2 Department of Earth and Environmental Sciences, Geography and Tourism Research Group, KU Leuven, Heverlee, Belgium; 3 Department of Geography and Resource Management, The Chinese University of Hong Kong, Hong Kong SAR, China; 4 Department of Geography, Ghent University, Ghent, Belgium; 5 Department of Urban and Regional Development Studies, Nicolaus Copernicus University, Torun, Poland

**Keywords:** High-speed rail, Technology-intensive manufacturing, Yangtze River Delta, Staggered difference-in-difference, Spatial Durbin model, Environmental social sciences, Socioeconomic scenarios, Sustainability

## Abstract

High-speed rail (HSR) may influence economic activities that rely heavily on innovation by facilitating skilled labour, face-to-face interactions, and knowledge spillovers. This study explores how HSR development affects the spatial distribution of technology-intensive manufacturing (TIM) in the Yangtze River Delta (YRD), China. Using a panel dataset including 24 cities for the period 2007–2016 and employing the output of communications equipment, computers, and other electronic equipment (CCOE) as a proxy for TIM’s economic productivity at the city level, we apply the staggered difference-in-differences (DID) and spatial Durbin model (SDM) to measure the impacts of HSR’s initial opening and connectivity on CCOE development and capture the spatial spillover effects of HSR connectivity. Our findings indicate that the initial opening of HSR and HSR connectivity are negatively associated with CCOE productivity in both DID and SDM. Additionally, the reduction of CCOE is more pronounced in cities with larger populations and higher levels of economy. Moreover, HSR has a more significant effect on CCOE than other manufacturing sectors. However, the spillover effects remain insignificant, indicating HSR’s limited impact on CCOE development in adjacent cities within the YRD.

## Introduction

Technology-intensive manufacturing (TIM)^1,2^, also known as “high-tech manufacturing”, highlights the use of “advanced and cutting edge technologies (p. 18)^3^” in the manufacturing process. In this sense, innovation is important for TIM firms to gain competitive advantages^2^. The development of TIM has been foregrounded in China’s development policies. For example, the “Made-in-China 2025” policy implemented by the central government aims to promote industrial upgrading towards knowledge-intensive manufacturing and enhance product quality (p.66)^4^. In the Yangtze River Delta (YRD), the importance of TIM sectors, such as electronic and computer information, has been emphasized in various government policies in the last two decades^5^.

Geographical distance matters for innovation^6,7^. For example, technological exchanges may be embedded in the movement of human capital, especially skilled labour^5,8^ as well as face-to-face interactions^9,10^. Transport infrastructure, especially high-speed rail (HSR), significantly changes travel time and patterns, potentially playing an essential role in facilitating face-to-face interactions, inducing labour movement, and reshaping the spatial distribution of TIM^11,12^.

Existing studies have explored the impacts of HSR on economic performance and spatial distribution of industries such as service and manufacturing at large^13–15^. However, due to the uneven reliance on HSR, examining such effects across sub-sectors seems relevant to provide a more in-depth understanding. For example, Yang et al.^15^ found that the producer service industry concentrated in cities connected to HSR, while HSR had no significant effect on consumer and public service industries as the latter are more dependent on the size and nature of the local market. Furthermore, the relationship between HSR and innovation could be investigated beyond the common measures of patents^16,17^ and considering potential spatial spillover effects^7,18^.

Against this background, this paper investigates the relationship between changes in cities’ HSR and the corresponding shifts in their TIM sectors and those of adjacent cities. Specifically, we draw on the output of communications equipment, computers, and other electronic equipment (CCOE) as a proxy for TIM in China’s Yangtze River Delta from 2007 to 2016 to construct a city-level panel database. We employ the staggered difference-in-difference and spatial Durbin models to assess the impacts of HSR’s initial opening and connectivity on CCOE output, as well as potential spillover effects. The remainder of this paper is organized as follows. "Literature review" provides an overview of the relevant research and outlines the hypotheses, followed by an elaboration of data and methods. "Empirical results" presents empirical findings. "Conclusion and discussion" summarises the main conclusions, policy implications and research limitations.

### Literature review

#### The impacts of HSR on innovation and economic activities

Transport infrastructure may be related to the spatial distribution of firms through travel costs and mobility of production factors^19,20^. HSR mainly serves as a transport infrastructure for passengers, which may lead to a more significant impact on service industries^15,21^. For example, Han et al.^22^ found an association between Shinkansen expansion and real estate and commercial development in Japan. In China, Shao et al.^23^ observed different impacts of HSR’s opening on producer, consumer and public services. Furthermore, Jin and Ou^24^ found varying effects of HSR across producer service subsectors and highlighted rising production costs as a potential cause. On the other hand, in the analysis of large manufacturing firms in China’s Greater Bay Area, Chang et al.^13^ found that HSR opening is associated with a reduction of manufacturing firms and employees in individual counties. Related studies have also explored the various channels (e.g., market potential and human capital) through which the impacts of HSR on manufacturing may take place^25,26^.

Drawing upon Chen and Haynes’s framework^27^ and synthesizing from the existing literature^7,17,28^, several pathways between HSR development, innovation, and technology can be highlighted. First, HSR can facilitate the movement of skilled labour into individual cities and subsequently improve firms’ innovation capacity^27,29,30^. For example, existing studies have associated the opening of HSR with varying degrees of employment specialization across subsectors and regions^28,31^. Fritsch and Slavtchev^32^ have also suggested positive effects of specialization of TIM in innovation. Moreover, the significantly reduced travel time by HSR may facilitate knowledge transfers and provide access to advanced technology through face-to-face interactions and collaborations among skilled labour^7,17,33^. At the same time, there may be “agglomeration shadow” effects (p.1090)^34^, where individual cities’ labour force may be drawn away from economically less developed cities as transport connectivity improves^35^. Second, HSR may facilitate information exchanges and mitigate information asymmetry among firms, such as those between venture capital (VC) companies and innovative enterprises, thus promoting innovation in HSR-connected cities^36^. In addition, Duan et al.^37^ have associated HSR connections with VC and found variations in such effects across city size and industry sectors. Third, HSR may extend the spatial extent of suppliers, customers, and labour markets^27,38^. Assuming other factors remain constant, broader market access may be associated with promoting innovation^39^ and increased productivity^40^. Our hypotheses 1 and 2 are, therefore:

***Hypothesis 1: HSR is positively associated with growth in both technology-intensive manufacturing and other manufacturing sectors.***

***Hypothesis 2: The impacts of HSR on growth in technology-intensive manufacturing are greater in magnitude than those in other manufacturing sectors.***

#### Spatial patterns of HSR impacts

The impacts of HSR vary across space^27,41^. On the one hand, Chen and Haynes^27^ found that HSR accessibility had more substantial impacts in economically less developed regions than in developed ones, thus contributing to economic convergence. Yao et al.^42^ pointed out that HSR can facilitate the outflow of production factors from economically advanced cities, thus narrowing development gaps across cities. Additionally, nearby cities tend to benefit from cities with HSR connections through diffusion. For example, HSR facilitated the movement of resources and economic activities from economically developed cities to surrounding cities^43^. Yang et al.^18^ (p. 1) found that “the innovation spillover range of innovation cities to non-innovation cities is 300 km”. Therefore, the spatial diffusion of production factors by HSR may reshape regional economies' distribution and promote more balanced development.

On the other hand, the “siphon effect (p. A2)^44^” may result in disparities among cities^27^. Enhanced accessibility and connectivity after being connected with HSR can make production factors more easily attracted to economically advanced cities^35,45^. For example, Xu and Sun^35^ found that HSR connections can facilitate migration from economically less developed cities to developed cities. Particularly, skilled labour tends to be attracted to cities located in the economically more developed eastern provinces and with relatively large populations through HSR in China^29^. Furthermore, such an effect exists between HSR-connected cities and their adjacent cities as well. Dong (p. 603)^38^ found that “HSR promoted growth in the cities it directly passed through by drawing activities away from their neighbours”. Based on these analyses, we propose our third hypothesis:

***Hypothesis 3: HSR’s impacts on technology-intensive manufacturing have a positive spillover effect on adjacent cities.***

#### Methodological considerations of evaluating HSR impacts

The difference-in-difference (DID) model has been widely employed to investigate the socioeconomic impacts of HSR^15,46,47^. This method helps capture the differences with and without HSR and those before and after HSR operation^48,49^. However, the conventional two-way fixed effects based DID usually tests treated effects operated at the same time^50^, while the opening times of HSR vary across cities. Moreover, changes in HSR networks are often measured as continuous variables which cannot be tested in a conventional DID model^51^. Furthermore, incorporating the spatial spillover effects of HSR in DID models can pose challenges^15^. Aiming at a more comprehensive understanding of HSR’s impacts, it may be useful to employ the newly proposed staggered DID models^52,53^ to account for the varying opening time in different cities^50,54,55^ and adopt the spatial econometric models^56–58^ to incorporate continuous variables such as HSR connectivity and capture the potential spillover effects^59^.

## Research design

### Study area and data

The Yangtze River Delta (YRD) in this study consists of the centrally administrated municipality of Shanghai as well as Anhui, Jiangsu, and Zhejiang provinces^60^. The YRD has seen major development in TIM and HSR in recent decades, making the region a suitable case for assessing the interactions between TIM and HSR^5^. In the past two decades, local governments and the central government launched policies to promote high-tech industry development such as electronic and computer industries^5^. Specifically, in 2018, TIM output accounted for 40% of the YRD’s gross manufacturing outputs, which is twice as much as in 2008^8^. By late 2020, there were over 6000 km of HSR tracks in YRD^61^ and all prefectural level and above cities, except for Zhoushan, are connected.

**Figure. 1:**
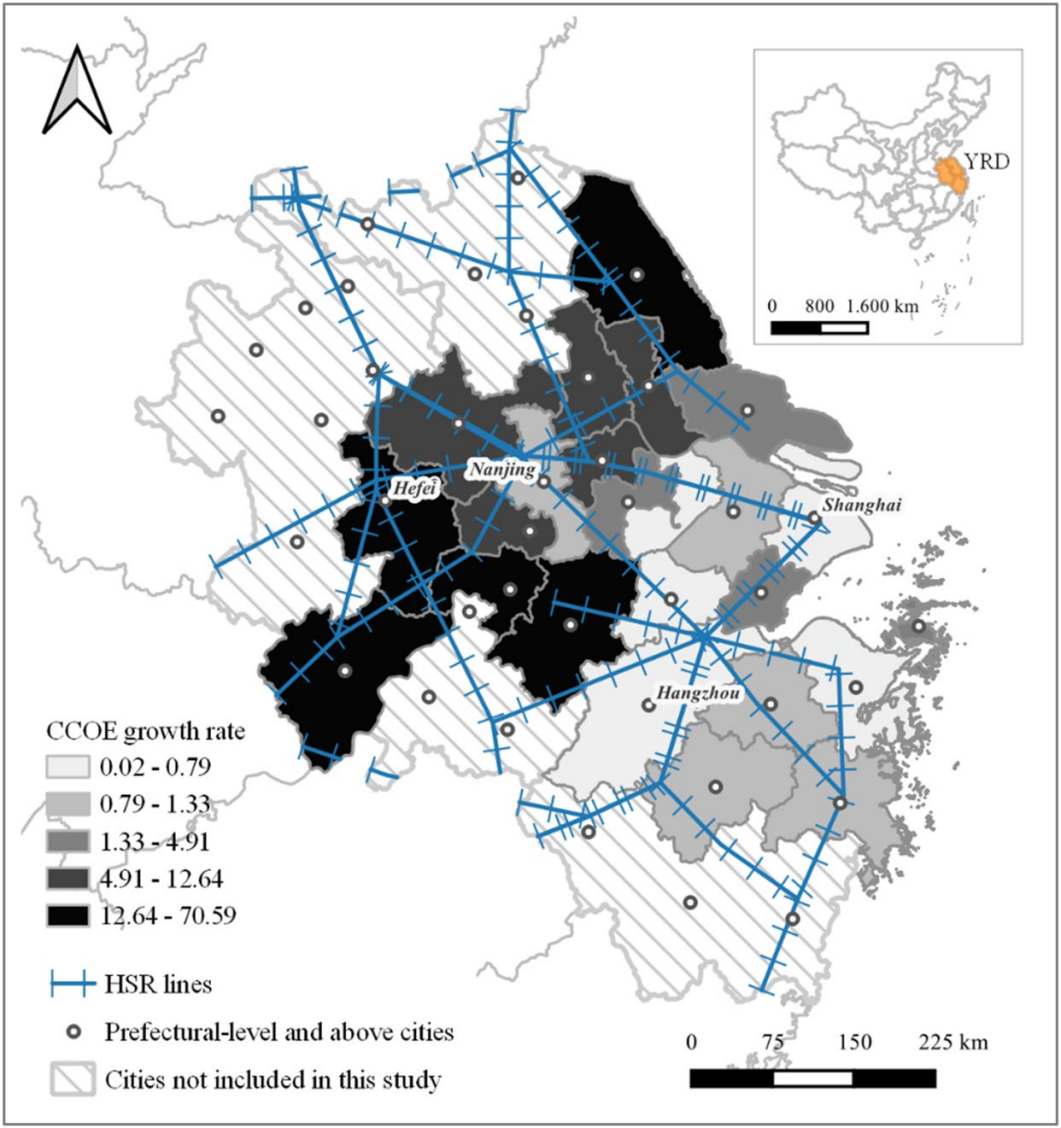
HSR network layout and growth rate of CCOE in YRD between 2007 and 2016 (Data source: China City Statistical Yearbook and statistical yearbooks of individual cities). QGIS 3.22.13: https://qgis.org/en/site/index.html.

In our study, we use the output of communications equipment, computers, and other electronic equipment sectors (CCOE) as a proxy for TIM’s development at the city level. CCOE was chosen due to its relatively high levels of R&D investment in the manufacturing sector^62^. According to a report from the Ministry of Science and Technology of the People's Republic of China, the amount of transaction contracts in CCOE ranked first among other high-tech manufacturing subsectors in 2019, accounting for approximately 25% of total technology transaction contracts^63^. Due to data availability, particularly in CCOE statistics, we constructed a database covering 24 out of 41 cities in the YRD (Fig. 1), above all representing the region’s more advanced cities^64^. By comparison, our analysis covers 24 out of the 26 cities included in an earlier version of the YRD regional plan^24,65^. In addition, we include the output of the aggregated manufacturing without CCOE as another dependent variable to analyse the impacts of HSR across sub-sectors.

**Figure. 2:**
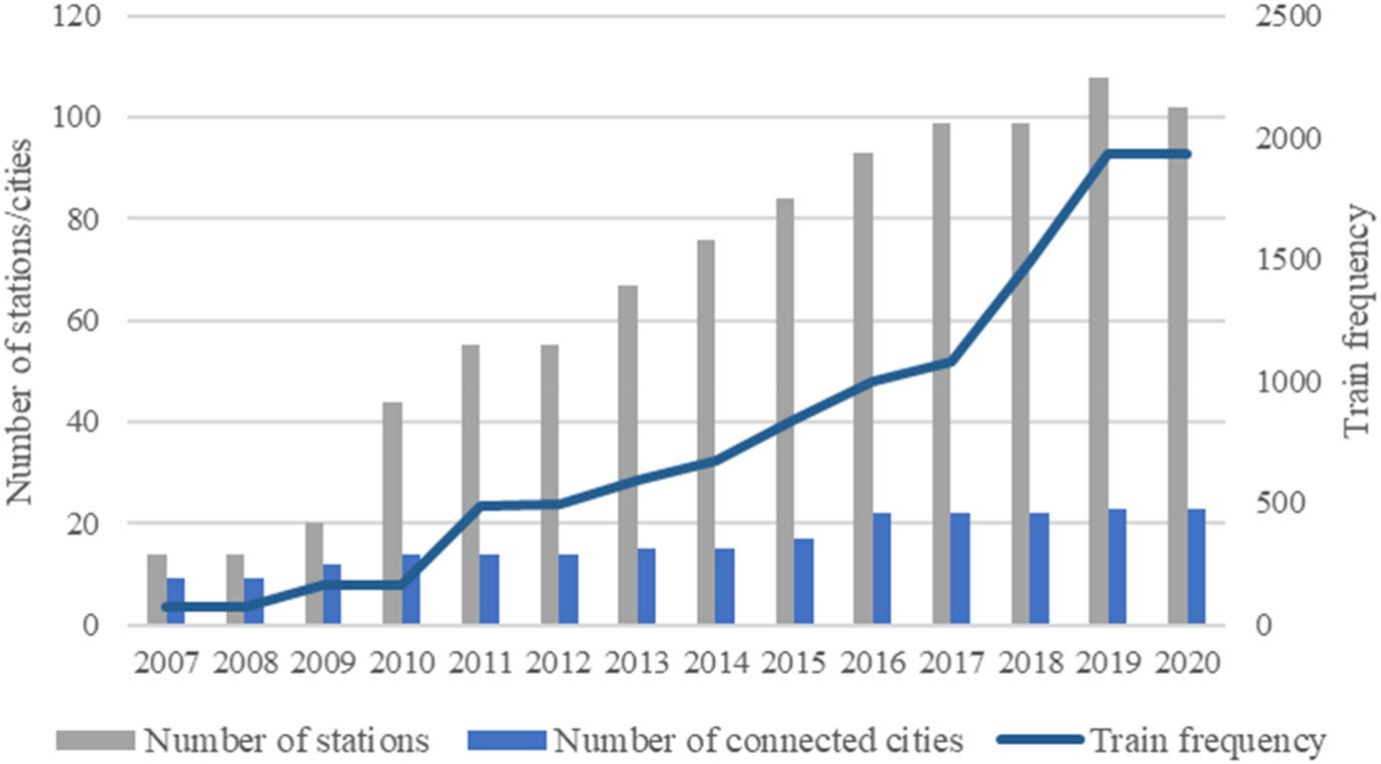
HSR development in the YRD (Data source: Passenger Train Timetable).

We choose 2007–2016 as our study period, with the start and end years chosen based on HSR development and expansion phases in China^66,67^. Furthermore, the number of HSR stations and connected cities in the study area stabilized after 2017 (Fig. 2). In addition, studies exploring transport’s impact on firm (re)location often adopt a timeframe of around ten years^13,68^.

Data on industrial statistics are mainly obtained from the China City Statistical Yearbooks and supplemented by statistical yearbooks of individual cities. Information on HSR is collected from the official Passenger Train Timetable released by China Railway Publishing House. City-level control variables are collected from China City Statistical Yearbooks.

### Variables

The first independent variable is a dummy variable (*Treat*_*it*_), with *Treat*_*it*_ set to 1 if city i is connected to the HSR network in year t and 0 otherwise. Jiao et al.^69^ emphasized the significance of incorporating both accessibility and connectivity into the assessment of potential HSR impacts. Similarly, some studies have employed continuous variables such as train frequency, accessibility, and connectivity, rather than only focusing on whether individual cities are connected^12,19,70^. Therefore, we adopt weighted degree centrality (*WDC*)^69^ as the second independent variable:

1 $$\begin{array}{c}{WDC}_{i}={k}_{i}^{\alpha }\cdot {s}_{i}^{1-\alpha }\end{array}$$

where *WDC*_*i*_ denotes city i’s weighted degree centrality, k_i_ represents the number of cities with direct HSR connections to city i, and s_i_ represents train frequency in city i. The parameter α is set to 0.5, following Jiao and colleagues^69,71^. A higher weighted degree centrality entails wider and more frequent connections through the HSR network, and vice versa^69^.

The average *WDC* has surged from 4.80 to 136.61 while the coefficient of variation for *WDC* decreased from 0.84 to 0.57. Our results indicate a significant HSR development and a more balanced distribution of HSR connectivity across cities during our study period (cf. Huang and Zong^72^). Furthermore, the two dependent variables exhibit spatial dependence, as evidenced by positive and statistically significant Moran’s I values^66^.

Following relevant studies^8,11,67^, we include the following control variables: population (ln(*Pop*)), economic level (ln(*PGDP*)), governmental support (ln(*ST*)), highway passenger volume (ln(*HPV*)), airport (*Airport*), and industrial structure (ln(*Ter*)). Table 1 summarises variable definitions and descriptive statistics. We note that there were adjustments of administrative boundaries during the study period. For example, the prefectural-level city Chaohu was split, with its four counties and one district merging into neighbouring cities of Hefei, Maanshan, and Wuhu in 2011. Still, Zongyang county was extracted from the prefectural-level city Anqing and merged into another prefectural-level city Tongling in 2015. Practically, we use the administrative boundaries of 2015 to re-calculate concerned observations. Specifically, variables for Heifei, Maanshan, and Wuhu from 2007 to 2010, and variables for Anqing in 2016 have been calibrated based on district and county-level statistics. Variables without district- and county-level statistics are split based on proximate weights^73^. For example, CCOE and other manufacturing outputs for Chaohu are split according to GDP of the constituting district and counties and highway passenger values are split based on district/county-level population.

**Table 1:** Statistical description of variables.

Variable	Definition of the variable	Obs.	Mean	Std. Dev	Min	Max
Dependent variables						
ln(CCOE)	ln (CCOE output)	240	23.522	2.096	18.061	27.626
ln(Man)	ln (output of other manufacturing sectors except for CCOE)	240	26.876	0.916	24.410	28.621
Independent variables						
Treat	A dummy on whether a city is connected with HSR	240	0.588	0.493	0	1
ln(WDC)	ln (weighted degree centrality + 1)	240	2.362	2.129	0	5.823
Control variables						
ln(Pop)	ln (city population)	240	15.341	0.526	13.782	16.490
ln(PGDP)	ln (GDP per capita)	240	11.003	0.671	9.171	12.338
ln(ST)	ln (finance expenditure for science and technology)	240	20.709	1.337	16.075	24.255
ln(HPV)	ln (highway passenger volume)	240	18.500	0.727	17.020	20.351
Airport	A dummy on whether there is at least one airport in a city	240	0.563	0.497	0	1
ln(Ter)	ln (tertiary industry output)	240	25.451	1.034	23.357	28.307

### Empirical models

#### Staggered difference-in-difference model

**Fig. 3:**
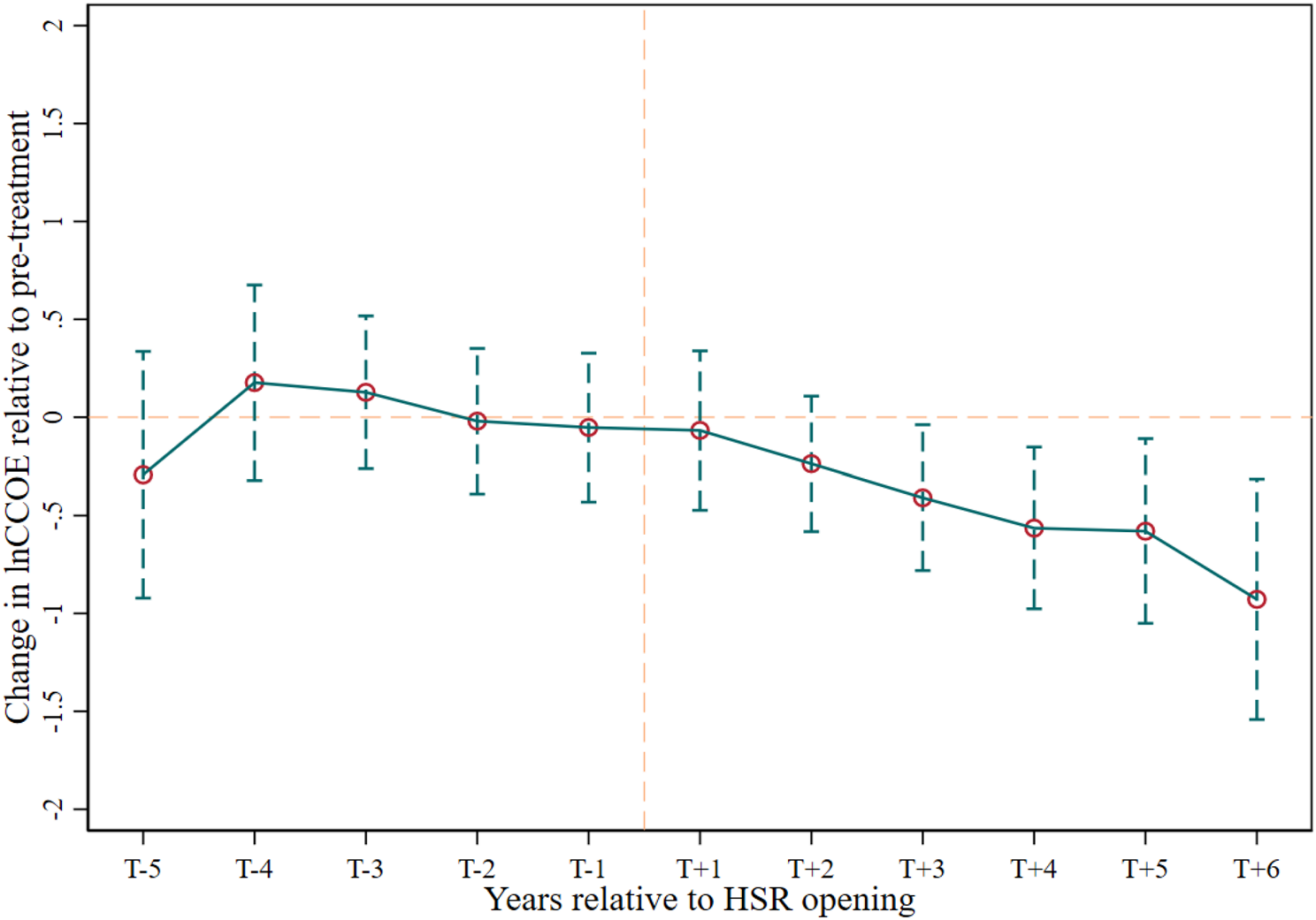
Parallel tends test: the differences in estimated coefficients of HSR impacts on ln(CCOE) relative to the reference year.

We employ the staggered DID^52,53^ to examine the impacts of HSR opening on TIM and other manufacturing sectors due to the varying opening times of HSR service across different cities. A Bacon decomposition test indicates a 36.24% of timing groups and implies potential bias in estimations with two-way fixed effects^74^. We therefore employ the Mundlak approach^53^ and the staggered DID regression model employed in our study is:

2 $$\begin{array}{c}ln({CCOE}_{it})={\alpha }_{0}+\beta {Treat}_{it}+{\mu }_{i}+{\vartheta }_{t}+{\varepsilon }_{it}\end{array}$$

3 $$\begin{array}{c}ln({Man}_{it})={\alpha }_{0}+\beta {Treat}_{it}+{\mu }_{i}+{\vartheta }_{t}+{\varepsilon }_{it}\end{array}$$

where ln(*CCOE*_*it*_*)* and ln(*Man*_*it*_*)* denote the logged values of COOE and other manufacturing sectors’ output of city i in year t, respectively. Parameter *β* reflects the average treatment effect of the HSR on CCOE and other manufacturing sectors. Both the city-fixed effect (*μ*_*i*_) and time-fixed effect (*ϑ*_*t*_) are included. *ε*_*it*_ is the error term. Change in ln(*CCOE*_*it*_*)* (see Fig. 3) has satisfied the parallel trend test^14,23^.

#### Spatial Durbin model

We apply spatial econometric models to assess the impacts of HSR connectivity on TIM and identify spatial spillovers. Our model selection follows the considerations proposed by Anselin^56^, LeSage and Pace^57^, and Elhorst and Vega^58^ and adopts the practical procedures introduced by Belotti et al.^75^. Specifically, the Likelihood-ratio (LR) test suggested that an individual fixed effect within SDM is appropriate. In addition, the results of the Wald test for θ = 0 and θ = − ρβ were significantly positive. Therefore, we adopt the SDM with city-fixed effect:

$$\text{ln}({CCOE}_{it})={\alpha }_{0}+\rho {\sum }_{j}{W}_{ij}\text{ln}{(CCOE}_{jt})+\beta {\text{ln}(WDC}_{it})+\theta {\sum }_{j}{W}_{ij}\text{ln}({WDC}_{it})$$

4 $$\begin{array}{c}+\delta {X}_{it}+\varphi {\sum }_{j}{W}_{ij}{X}_{jt}+{\mu }_{i}+{\varepsilon }_{it}\end{array}$$

$$\text{ln}({Man}_{it})={\alpha }_{0}+\rho {\sum }_{j}{W}_{ij}\text{ln}{(Man}_{jt})+\beta {\text{ln}(WDC}_{it})+\theta {\sum }_{j}{W}_{ij}\text{ln}({WDC}_{it})$$

5 $$\begin{array}{c}+\delta {X}_{it}+\varphi {\sum }_{j}{W}_{ij}{X}_{jt}+{\mu }_{i}+{\varepsilon }_{it}\end{array}$$

where ln(*CCOE*_*it*_) and ln*(Man*_*it*_) denote the logged values of COOE, and other manufacturing sectors' output of city i in year t, respectively. *ln(WDC*_*it*_*)* represents the logged value of weighted degree centrality. Parameters *ρ* and *θ* denote the spatial autoregressive coefficient of dependent and independent variables, respectively. *μ*_*i*_ and *ε*_*it*_ are the city-fixed effect and error term, respectively. The spatial weight matrix *W*_*ij*_ is based on rook contiguity and is row-normalized. In this matrix, Zhoushan is treated as an adjacent city of Ningbo to avoid isolation.

We follow previous studies to interpret our independent variables' direct and indirect effects^30,76^. More specifically, direct effects characterize the relationship between independent and dependent variables of the same cities, while indirect effects capture how independent variables in individual cities associate with dependent variables of their adjacent cities. We employ *jwdid*^51^ and *xsmle*^74^ packages in Stata to estimate DID and SDM models, respectively.

## Empirical results

### Impacts of HSR on individual cities’ CCOE

The regression results of the initial opening of HSR on CCOE and other manufacturing outputs are shown in Table 2. The coefficient of *Treat* is significantly negative for CCOE, suggesting that cities with HSR opening may be associated with less CCOE growth rate^77^. The coefficient of *Treat* on other manufacturing is insignificant and negative.

**Table 2:** Estimation results of the initial opening of HSR service’s impacts on different sectors based on staggered DID model.

	ln(CCOE)	ln(Man)
	(1)	(2)
Treat	− 0.630	− 0.149
95% CI	[− 1.111 to − 0.149]	[− 0.369 to 0.071]
p-value	0.010	0.185
Control variables	No	No
City-fixed effect	Yes	Yes
Time-fixed effect	Yes	Yes
Obs	240	240

Table 3 summarises the estimation results based on SDM with city-fixed effects. Columns (1) and (2) are the results of CCOE and other manufacturing sectors with control variables, respectively. The direct effect results in the SDM model show consistency with those in the staggered DID model. Specifically, the direct effect of ln(*WDC*) on the *CCOE* variable is – 0.073 and significant at the 5% level, implying that an average 1% increase in HSR connectivity leads to a 0.073% decline in CCOE output. Based on these results, both HSR’s initial opening and HSR connectivity have significantly negative impacts on CCOE output, and we therefore reject Hypothesis 1.

**Table 3:** Direct and indirect effects of connectivity on different sectors based on SDM.

	Ln(CCOE)	ln(Man)
	(1)	(2)
Direct effect		
ln(WDC)	− 0.073** (0.033)	0.000 (0.012)
ln(Pop)	2.892** (1.340)	0.677** (0.336)
ln(PGDP)	3.333*** (1.242)	1.241*** (0.252)
ln(ST)	0.450*** (0.129)	0.099*** (0.028)
ln(HPV)	0.161 (0.099)	0.076** (0.038)
Airport	0.137 (0.183)	0.009 (0.049)
ln(Ter)	− 1.451 (1.070)	− 0.207 (0.250)
Indirect effect		
ln(WDC)	0.094 (0.087)	− 0.026 (0.021)
ln(Pop)	1.955 (2.038)	1.046* (0.563)
ln(PGDP)	1.082 (1.581)	1.123*** (0.355)
ln(ST)	− 0.518*** (0.158)	− 0.077* (0.042)
ln(HPV)	− 0.180 (0.218)	− 0.094* (0.053)
Airport	− 0.534 (1.250)	0.527* (0.306)
ln(Ter)	− 0.844 (1.502)	− 0.885*** (0.325)
Spatial rho	0.419*** (0.099)	0.258** (0.114)
City-fixed effect	Yes	Yes
Obs	240	240
R-squared	0.815	0.890

*p < 0.10, **p < 0.05, ***p < 0.01; the values in parentheses are standard errors.

The negative impacts may be related to the following factors. First, as the sample in our study primarily consists of economically more advanced cities, this may partly explain the observed CCOE reduction, e.g., production costs are already high in cities with large GDP and populations^64,78^. Second, HSR connections may be conducive to increases in labour and land costs^79,80^ and facilitate the mobility of production factors^43^. Improved HSR connectivity and increasing production costs may drive firms away from these cities^81,82^. Similarly, Wu et al.^78^ highlighted rising production costs as a driving force behind industrial relocation away from economically developed regions in YRD. Liu et al.^31^ also found ‘constraining’ effects of HSR on high-end Pearl River Delta manufacturing, citing increased housing prices as a possible cause. Still, Chang et al.^13^ reported that employment in large-scale manufacturing was reduced by 12.2% in China’s Great Bay Area after implementing an HSR connection. This may also be in line with Baum-snow et al.’s observation^83^ that radial railroads in China caused declines in manufacturing activities and employment in economically more developed cities. Third, HSR may be associated with labour movement. For example, Feng et al.^29^ found that HSR’s positive effect on the outflow of skilled labour, especially in eastern China and cities with more than 1 million population.

Meanwhile, the direct effects of ln(*WDC*) in other manufacturing sectors remain insignificant. In addition, the absolute magnitude of HSR impacts is greater in CCOE than in other manufacturing sectors in both models. Therefore, our results suggest that the association of HSR with CCOE is more significant than that for other manufacturing sectors, supporting Hypothesis 2. The results may be attributed to the fact that the use of rail by other manufacturing sectors focuses on moving freight, while HSR is primarily served for personal travel^15^.

Based on the results in Table 3, we explain the effects of main control variables on CCOE development. The direct effect of ln(*Pop*) on CCOE is positively significant at the 5% level, indicating that CCOE growth is more associated with populous cities. The direct impacts of ln(*PGDP*) on CCOE are positively significant at the 1% level, suggesting that CCOE output is associated with higher levels of economic development. The direct effect of ln (*ST*)) is positively significant at the 1% level, reflecting the importance of governmental support in TIM development. These observations align with the conjecture that these production factors may stimulate innovation and economic growth in high-tech industries^5,84^. Nevertheless, the direct effects of ln(*HPV*) and *Airport* are insignificant. This may be related to the fact that only one airport opened during our research period, potentially limiting the ensuing ability to attract talent.

### Impacts of HSR on adjacent cities’ CCOE

Table 3 also presents the indirect effects of all variables based on SDM, reflecting the relationship between HSR connectivity and CCOE output in adjacent cities. The indirect effect of ln(*WDC*) on CCOE is positive but insignificant. Thus, Hypothesis 3 is rejected. This may be due to the fact overall the study area is relatively more developed within the Chinese context and well-served with HSR connections^64,85^. In addition, Wu et al.^78^ suggested that the relocation of manufacturers may not always be limited within the YRD, thus going beyond the scope of the current analysis.

The indirect effect of ln(*ST*) is statistically significant and negative, with a coefficient of − 0.518. On average, every 1% science and technology expenditure growth will reduce the CCOE output in their adjacent cities by 0.518%. This may be in line with the observation that local governments’ supports for science and technology are critical for the development of high-tech industries^12^.

### The interaction effects on CCOE

We examine the effects of interaction terms on the development of CCOE to assess the potential interactions between HSR and socio-economic variables as well as other transport infrastructures (Table 4). The direct effect of ln(*WDC*) is statistically significant and negative, while the direct effects of ln(*Pop*) and ln(*PGDP*) are statistically significant and positive (Table 3). The direct effect of the two interaction variables (ln(*WDC*)*ln(*Pop*) and ln(*WDC*)*ln(*PGDP*)) are statistically significant and negative (see Table 4). This suggests that HSR connectivity’s negative impacts on CCOE are more likely to be observed in cities with higher levels of per capita GDP and population.

**Table 4:** Estimation results of impacts of interaction terms on CCOE based on SDM.

	(1)	(2)	(3)	(4)	(5)	(6)
Direct effect						
ln(WDC)	3.200* (1.915)	1.891*** (0.466)	1.452*** (0.461)	1.228*** (0.461)	− 0.023 (0.060)	3.343*** (0.649)
ln(Pop)	4.485*** (1.459)	2.739** (1.242)	3.389*** (1.255)	3.003** (1.280)	3.487** (1.394)	3.954*** (1.198)
ln(PGDP)	3.955*** (1.192)	2.848*** (1.097)	3.107*** (1.163)	3.394*** (1.185)	3.643*** (1.349)	3.303*** (1.006)
ln(ST)	0.362*** (0.113)	0.311** (0.123)	0.437*** (0.108)	0.455*** (0.130)	0.367*** (0.116)	0.256** (0.109)
ln(HPV)	0.197** (0.087)	0.005 (0.056)	0.045 (0.066)	0.349*** (0.126)	0.150* (0.091)	0.062 (0.059)
Airport	0.068 (0.187)	− 0.288 (0.206)	− 0.134 (0.209)	0.182 (0.180)	0.100 (0.197)	− 0.327* (0.191)
ln(Ter)	− 1.785* (0.968)	− 0.535 (0.974)	− 0.700 (0.993)	− 1.218 (1.073)	− 1.740 (1.121)	− 0.890 (0.938)
ln(WDC)*ln(Pop)	− 0.215* (0.125)					
ln(WDC)*ln(PGDP)		− 0.180*** (0.042)				
ln(WDC)*ln(ST)			− 0.074*** (0.021)			
ln(WDC)*ln(HPV)				− 0.071** (0.032)		
ln(WDC)*Airport					− 0.118 (0.080)	
ln(WDC)*ln(Ter)						− 0.135*** (0.025)
Indirect effect						
ln(WDC)	2.352 (4.230)	1.947* (1.030)	0.129 (1.031)	0.341 (1.659)	0.099 (0.118)	4.489*** (1.379)
ln(Pop)	1.553 (2.133)	2.613 (1.719)	2.019 (1.988)	1.670 (2.140)	1.470 (2.155)	2.594 (1.599)
ln(PGDP)	0.374 (1.389)	0.783 (1.286)	0.309 (1.544)	1.154 (1.567)	0.754 (1.507)	0.247 (1.158)
ln(ST)	− 0.430*** (0.153)	− 0.634*** (0.111)	− 0.452*** (0.139)	− 0.552*** (0.150)	− 0.392** (0.169)	− 0.608*** (0.115)
ln(HPV)	− 0.136 (0.188)	− 0.050 (0.183)	− 0.009 (0.223)	− 0.109 (0.308)	− 0.186 (0.226)	− 0.097 (0.161)
Airport	− 0.848 (1.162)	− 1.284 (1.364)	− 0.868 (1.368)	− 0.759 (1.211)	− 0.950 (1.279)	− 1.535 (1.187)
ln(Ter)	− 0.375 (1.211)	0.058 (1.304)	− 0.638 (1.526)	− 1.128 (1.503)	− 0.520 (1.400)	0.592 (1.166)
ln(WDC)*ln(Pop)	− 0.146 (0.276)					
ln(WDC)*ln(PGDP)		− 0.169* (0.093)				
ln(WDC)*ln(ST)			− 0.000 (0.048)			
ln(WDC)*ln(HPV)				− 0.015 (0.109)		
ln(WDC)*Airport					− 0.031 (0.209)	
ln(WDC)*ln(Ter)						− 0.172*** (0.054)
Spatial rho	0.440*** (0.093)	0.340*** (0.061)	0.477*** (0.090)	0.410*** (0.091)	0.449*** (0.083)	0.284*** (0.060)
City-fixed effect	Yes	Yes	Yes	Yes	Yes	Yes
Obs	240	240	240	240	240	240
R-squared	0.814	0.800	0.816	0.812	0.828	0.785

*p < 0.10, **p < 0.05, ***p < 0.01; the values in parentheses are standard errors.

The direct effect of ln(*HPV*) is positive but insignificant (Table 3), and the direct effect of ln(*WDC*)*ln(*HPV*) is statistically significant and negative at the 5% level (Table 4). Nevertheless, the result of ln(*WDC*)*Airport remains insignificant, which may be related to relatively small changes in air travel as mentioned in "Impacts of HSR on individual cities’ CCOE".

## Conclusion and discussion

This paper explored the effects of HSR development on TIM, using CCOE output as a proxy for TIM economic productivity and focusing on 24 cities in the YRD from 2007 to 2016. Employing the staggered DID and SDM, results show that the initial opening of HSR and inter-city connectivity brought by HSR negatively impact CCOE output. Such negative impacts become more pronounced in cities with larger populations, and relatively higher levels of economic development. Additionally, the association of HSR with CCOE are more significant than those of other manufacturing sectors. Based on the spillover effects in SDM, we did not observe significant results, indicating the limited effect of HSR on CCOE development in adjacent cities within the YRD.

The key findings of this paper lead us to the following policy implications. First, our results point to negative impacts of HSR on CCOE outputs. The association between HSR and CCOE differs from the association between HSR and other manufacturing sectors. Future transport and industrial policies may therefore consider such varied associations. Second, the current analysis does not identify significant spillovers of HSR in the YRD. However, given the potential significance of HSR’s spatial effect^29,43^, future studies may unpack potential spatial spillovers. This is especially relevant given that the development of HSR, innovation and industry in China may be affected by both the market and state^86^ and notably inter-city competition^5,87–89^.

Other limitations of the current study include the following. First, our study area is constrained to economically developed cities due to data availability. Extending the geographical scope to incorporate a larger sample may help to arrive at a more comprehensive understanding of HSR’s spatial impacts on TIM. Second, our analysis is confined to the city level and does not assess the impact mechanisms. A follow-up study could use firm-level data to capture the dynamics at more refined geographical levels as well as across industrial subsectors.

## Data Availability

The datasets analysed during the current study are available from the corresponding author on reasonable request.
